# Caregivers’ perception and acceptance of malaria vaccine for Children

**DOI:** 10.1371/journal.pone.0288686

**Published:** 2023-07-26

**Authors:** Victoria Bam, Abdulai Mohammed, Abigail Kusi-Amponsah, Jerry Armah, Alberta Yemotsoo Lomotey, Hayford Isaac Budu, Collins Atta Poku, Joana Kyei-Dompim, Catherine Dwumfour

**Affiliations:** 1 Department of Nursing, Kwame Nkrumah University of Science and Technology, Kumasi, Ghana; 2 Midwifery Training College, Tumu, Tumu Upper West Region, Ghana; 3 Department of Nursing Science, Faculty of Medicine, University of Turku, Turku, Finland; The University of Dodoma, UNITED REPUBLIC OF TANZANIA

## Abstract

**Background:**

Malaria is a disease of public health concern and in endemic areas, pregnant women and children under-five years are vulnerable to the disease. The introduction of the pilot program of a malaria vaccine for children under-five years in Ghana is an intervention to further reduce the burden of the disease. However, the availability of the vaccine does not necessarily mean it will be accepted by the public. This is why the perceptions and acceptance of the vaccine among mothers of these children are worth exploring.

**Method:**

A descriptive qualitative study, with the aid of a semi-structured interview guide, was utilized in collecting data from ten (10) purposively sampled mothers whose children were taking the malaria vaccine in a municipality in Ghana. Written informed consent was obtained from all participants. The audiotaped interviews were transcribed verbatim and inductively analyzed into themes describing their perceptions and acceptance.

**Results:**

Participants were aged between 22 and 40 years with eight (8) of them married. Three themes emerged from the study. "Awareness of malaria and the malaria vaccine" (1), “Insight into the malaria vaccine” (2), where participants communicated the beliefs and judgments formed on the vaccine, its benefits, and the need for vaccinating their children. With the third theme "Reaction to vaccine” (3), participants communicated their motivation to vaccinate their children and their concerns about the administration of the vaccine.

**Conclusion:**

The caregivers had positive perceptions about the malaria vaccine for children, with fewer hospital admissions and saving money as some benefits. Healthworkers played a significant role in influencing the acceptance of the vaccine. However, the fear of the unknown concerning the side effects of the vaccine serve as a possible barrier to recommending the vaccine to other caregivers. Health education must also address the fears of caregivers in order to enhance recommending the malaria vaccine to other caregivers and promote uptake of the vaccination.

## 1. Introduction

Malaria is a leading cause of death and illness among young children, hitting hardest in resource poor countries. The disease has consistently been reported to affect millions of people and annually kills hundreds of thousands of people, mostly African children under age five [1–4]. It is estimated that 247 million cases of malaria were reported globally in 2021, with 95% of this in the African region, whiles deaths caused by malaria are estimated at 619, 000, with children below 5 years accounting for over 80% of these deaths [1, 4]. Over 96% of malaria deaths occur in sub-Saharan Africa, out of which 61% to about 67% of the cases are children under the age of five years [4, 5]. The disease is also implicated in life-threatening conditions such as severe anemia, respiratory distress, shock, and coma [2].

Ghana is among 11 other countries that accounted for about 70% of the global burden of malaria in 2017 [3]. It is estimated that 20% of children in Ghana have malaria parasites in their blood and the disease causes almost 2,000 deaths yearly, with about 48% of these deaths being children under five years [6].

The existing fight against the disease is being waged on different grounds to include bednet distribution, indoor spraying, and chemotherapy. That apart, the development of resistance of the malaria parasite to drugs pose a challenge for better and cost-effective ways of curtailing the menace [1]. Recent innovations in technology and interventions such as rapid diagnostic tests, microscopic tests, and confirmatory diagnosis; the use of long-lasting insecticide nets, and mass campaigns have contributed a great deal to the decline of the malaria burden [7, 8]. However, the disease remains a threat due to drug and insecticide resistance [9–11]. Therefore, additional interventions like vaccines are needed to combine with the already existing interventions like indoor residual spraying and insecticide-impregnated bed nets that will target various stages of the malaria parasite to combat the threat [10]. Vaccines have been proven to be an efficacious means of preventing disease and death, hence the need for the new malaria vaccine (RTS, S also known as Mosquirix) as essential tools to eradicate malaria [1].

Ghana has achieved tremendous success in the fight against malaria through its existing interventions (medications targeting the parasite and vector control activities) by significantly reducing morbidity and mortality related to malaria [12]. However, malaria remains a major health threat as the country continues to experience a significant malaria disease burden [13]. Ghana, Kenya and Malawi have been part of a process to adopt the malaria vaccine as far back as 2006 with the conduct of Phase II studies in Ghana at the Kintampo Health Research Centre [14]. The implementation of RTS, S in the three countries is to augment other existing anti-malaria interventions to effectively control the menace [15].

The RTS, S is the first and only vaccine that has currently proven to significantly reduce malaria infection with an acceptable safety profile [16, 17]. The vaccine is intended to be delivered as part of the Expanded Programme on Immunizations (EPI). The EPI is one of the most efficient and high-performing programs in sub-Saharan Africa and Ghana has successfully introduced new vaccines into the EPI routine immunization schedules. For the malaria vaccine, children who present at the health facilities would be vaccinated within the recommended four (4) dose schedules and a child needs to continue with other malaria preventive practices for full protection.

The malaria vaccine program in Ghana is being implemented in six (6) selected regions namely Central, Volta, Ahafo, Bono, Bono East, and Upper East Regions. The pilot implementation of the malaria vaccine in the Upper East region is in the Kassena-Nankana East Municipality and Kassena-Nankana West District. In 2019, a total of 28,047 persons were diagnosed with malaria in the Kassena-Nankana Municipality. Children less than five (5) years accounted for over 90% of these morbidity cases. The mortality rate of children under-five years due to malaria stood at 38.2% in the municipality [18].

The effectiveness of vaccines depends on both their clinical efficacy and the knowledge and perceptions of the community about the vaccine [19] and the acceptance and compliance with vaccine have been known to be influenced by awareness as well as knowledge of the benefits of these vaccines [20, 21]. Lack of community support during a malaria vaccine promotion in Kintampo, Ghana due to inadequate information and attitudes led to poor community acceptance, while some rejected immunizations [22]. Because caregivers of children under- five years are the sole decision-makers on vaccine uptake for them, these children can only be immunized when their caregivers accept the use of the vaccines and are willing to comply with the vaccination programme for effective outcomes. Therefore, to guide policymakers’ actions toward successful vaccine implementation programs, it is worth exploring the perception and acceptance of caregivers about the malaria vaccine. The response from primary caregivers of the malaria vaccine intervention will help gain insight into their experiences of the pilot programme, and this will generate information that will be helpful to stakeholders for the roll-out of the programme. It is on this premise that the study was conducted.

## 2. Materials and methods

### 2.1 Design and setting

A descriptive qualitative approach was employed to understand the import and meaning that different participants attached to the issue [23]. The study was conducted in the Kassena-Nankana East Municipality because it is one of the two districts among the five regions in the Northern part of Ghana that is participating in the pilot implementation of the malaria vaccine programme initiated in May 2019 [24]. The municipality consists of seven (7) sub municipals namely Kologo, Pungu, Manyoro, Vunania, Navrongo Central, Navrongo East, and Wuru. Health facilities include a hospital located in the capital city of the Municipal, 2 Health Centres, 17 Community-Based Health Planning and Services (CHPS) compounds, a Health Research Centre, a Private Clinic, and a Health post. Kassena-Nankana East Municipality is located in the western part of the Upper East Region of Ghana and has Navrongo as its capital town. The Municipality is bounded by seven (7) districts and one country; to the North by Kassena Nankana West District and Burkina Faso, on the East by Kassena Nankana West District, Bolgatanga Municipality, Talensi District and Bongo District, to the West by the Builsa South and Builsa North Districts and on the South by West Mamprusi Municipality in the North East Region.

### 2.2 Sampling and sample size

The number of participants was determined by data saturation. A purposive sampling method was adopted to enroll ten (10) caregivers (Table 1) who met the inclusion criteria. The inclusion criteria were (1) Caregivers of children who were below five (5) years and taking the RTS, S vaccine at the time of the study, (2) Caregivers who spoke either English, Kassim, or the Nankan language, (3) Caregivers who were 18 years and above.

**Table 1:** Sample size.

NUMBER OF PERSONS RECRUITED	COMMUNITY	SUB-MUNICIPAL
1	Pungu	Pungu
1	Punyoro	
2	Kologo	Kologo
1	Zuo	
1	Vunania	Vunania
3	Navrongo town	Navrongo Central
1	Tano	

### 2.3 Data collection and analysis

The participants were from seven communities and the initial contact with them was in the four health centers in the Kassena-Nankana East Municipality. Fifty-nine (59) caregivers who met the inclusion criteria were approached by the researchers, however, data saturation was reached at the tenth participant when no new information was emerging from the interviews. Those that consented by signing a consent form after reading and interpreting the participants’ information sheet were made to decide on the venue and time convenient to them for the interview. A semi-structured interview guide, designed based on study objectives guided the data collection on perceptions and acceptance of the malaria vaccine and related factors. The data collection was from May to July 2021. Face-to-face interviews were conducted in the homes of all participants in 7 communities (Pungu, Punyoro, Kologo, Zuo, Vunania, Navrongo, Tano) in 4 sub-municipals (Pungu, Kologo, Vunania, Navrongo Central) (Table 1). Each interview lasted between 30 minutes to an hour, with four (4) interviews conducted in English and three each in the Kassim and Nankan languages. Prompts were used to elicit more information, notes were also taken during the interviews and non-verbal cues were noted.

The data were analyzed using the inductive thematic analysis approach. The audio recordings were transcribed verbatim, compared, and supplemented by notes that were taken during the interviews. Data that were collected in Kassim and Nankan were transcribed and translated into English and back to Kassim or Nankan to ensure credibility. The transcripts were read through several times and compared among researchers to ensure consistency in meanings. Key expressions were evaluated, and expressions with similar meanings were organized into codes. The codes were examined for patterns to generate the themes and sub-themes. The generated themes were compared with the transcripts (identified with pseudonyms) to ensure they represent what participants said.

### 2.4 Ethical consideration

Ethical approval was obtained from the Committee on Human Research, Publications and Ethics (CHRPE) of Kwame Nkrumah University of Science Technology (KNUST) with a reference number of “CHRPE/AP/157/21”. Approval was also sought from the Upper East Regional Health Directorate before the commencement of the study. Voluntary participation was ensured in the study. The purpose, risks, and benefits of the study were explained to each participant. Their rights to withdraw from the study at any point during the study were also explained to them. Written informed consent was obtained from all the participants before they were included in the study. Each of them either signed or thumb-printed an informed consent form before their data was collected. Participants were also given an information sheet detailing what they needed to know about the study before they were asked to sign the informed consent.

## 3. Results

### 3.1 Socio-demographic characteristics of participants

Participants were all females, aged between 22 and 40 years with a majority (n = 8) of them being married. Almost all the participants were Christians (n = 9). For the educational level of participants, a majority (n = 6) attained secondary education. Half (n = 5) of the participants were public/civil servants, and three (3) were engaged in their private businesses (Table 2).

**Table 2:** Sociodemographic characteristics of participants.

Variables	Frequency	Percentage (%)
Total participants	10	
**Age (years)**		
20–25	2	20
26–30	3	30
31–35	3	30
36–40	2	20
**Marital Status**		
Married	8	80
Single	1	10
Divorced	1	10
**Religion**		
Christianity	9	90
Islam	1	10
**Educational Level**		
Basic	1	10
Secondary	6	60
Tertiary	3	30
**Occupation**		
Unemployed	2	20
Private business	3	30
Public/civil servant	5	50

### 3.2 Themes and sub-themes

Three themes and eight (8) sub-themes were identified from the data collected. The themes include “Awareness of malaria and the malaria vaccine”, “Insight into the malaria vaccine” and “Reaction to the malaria vaccine”. The organization of the themes and their various sub-themes is represented in Table 3 below. In all the quotes to support themes and sub-themes, pseudonyms have been used for the participants.

**Table 3:** Themes and sub-themes.

Themes	Sub-themes
Awareness of malaria and the malaria vaccine	Information on malaria
	Information on the malaria vaccine
Insight into the malaria vaccine	Need for children to vaccinate
	Beliefs about the malaria vaccine
	Forming judgments on the vaccine
	Benefits of vaccinating a child
Reaction to the malaria vaccine	Motivation to vaccinate a child
	Concerns about vaccine administration

#### 3.2.1 Awareness of malaria and malaria vaccine

Participants’ level of awareness of the nature of malaria and the vaccine for malaria was explored by this theme. The results of the study show a widespread awareness and knowledge of both malaria and the malaria vaccine. Two subthemes; “Information on malaria” and “Information on malaria vaccine” emerged from this theme.

*3*.*2*.*1*.*1 Information on malaria*. Participants were able to point out that mosquito bites cause malaria and mentioned some cardinal signs and symptoms of the disease. They gave their source of information as mainly the health workers. Mass media as well as their parents at home were also sources of information on the disease. Also, participants pointed out that malaria affects everyone, however, the disease is severe in children. Examples of their narratives are the following quotes.

“*Malaria in children is caused and spread by a mosquito which carries a plasmodium from an infected person to an uninfected person*. *This plasmodium multiplies to generate symptoms like fever*, *cold*, *vomiting*, *fatigue*, *loss of appetite*, *and general body weakness*. *I heard about it from health workers”*. *(40-year-old Charity)*

“*…we were children when our parents started talking about malaria”*. *(35-year-old Lariba)*

*“I also read them in books, heard it on the radio, health talks at the health facility”**. (**34-year-old Anita)*

*3*.*2*.*1*.*2 Information on malaria vaccine*. Participants knew what vaccines are since their children had been receiving vaccination at the Child Welfare Clinic for some time. They demonstrated their knowledge of the malaria vaccine by pointing out that the vaccine is taken four times by children below the age of five years to protect them from getting malaria. This was evident in their quotes;

“*Vaccines are substances usually introduced into one’s body to stimulate the production of antibodies which provide immunity against a disease”(40-year-old Charity)*.

“*Vaccines are medicines given to children to protect them and make them healthy*. *The malaria vaccine is given four times to children to protect them against malaria” (27-year-old Jemimah)*.

*‘The malaria vaccine is given to children four times for a period of two years to prevent malaria infection in children*. *My child received some of the vaccines*” (*38-year-old Asana)*.

Some participants however said they did not have much information on it. An example is what Anita (34 years) said:

’*With the vaccine*, *I don’t have much information about it*. *I only know that when you vaccinate your child*, *he should not get malaria but when he gets it*, *I don’t know how it will affect him*. *I would wish that when you vaccinate*, *he should not get malaria again’ (34-year-old Anita)*.

#### 3.2.2 Insight into the malaria vaccine

Participants’ perceptions of the malaria vaccine were significant in their determination to consent for their children to be vaccinated. Data revealed that most participants view the malaria vaccine generally as a very potent protective agent, which when complemented with other interventions is capable of dealing with the malaria menace. Four (4) sub-themes, “Need for children to vaccinate”, "Beliefs about malaria vaccine", "Forming judgments on the vaccine" and "Benefits of vaccinating a child" emerged from this theme.

*3*.*2*.*2*.*1 Need for children to vaccinate*. Participants were of the view that vaccinating every child with the malaria vaccine was necessary considering the severity of the disease. They were certain that malaria could be prevented if parents/caretakers were encouraged in vaccinating every child as it is done with other vaccines like polio. Again, participants were motivated to allow their children to be vaccinated against malaria because of the severity of the condition, and the safety and efficacy of the malaria vaccine. They added that the malaria vaccine, just like almost all other vaccines may have some side effects which are self-limiting, and hence was safe. This was evident in the responses of participants.

“*After injecting my child with the vaccine for two years I think that the adverse effects of the vaccines are very minimal and there is the need for every child to be vaccinated against malaria to get them well protected”*. *(40-year-old Charity)*.

“*Vaccinating every child should be the priority of Ghana Health Service*. *I have noticed that the vaccine does not have any severe adverse effects except few mothers who complain about the high body temperature of their children after taking the vaccine*. *My son equally suffered similar side effects after taking the vaccine but everything was normal the following day*. *I think it is very safe for every child to take vaccine for protection” (22-year-old Jane)*.

*3*.*2*.*2*.*2 Beliefs about the malaria vaccine*. Cultural beliefs and religious affiliations of people play a major role in disease control programs as these shape their behavior and can sometimes influence their decision in either accepting or rejecting medical interventions. All participants in the study reported that there were no known cultural or religious beliefs in their communities that influence the decisions they make regarding the malaria vaccine. The following quotes are examples.

*‘I do not have or know of any cultural beliefs against taking vaccine’ (27-year-old Jemimah)*.

’*We do not have any cultural beliefs against the vaccine*. *We are Christians and nothing is preventing us from taking the vaccine’ (35-year-old Alberta)*.

*My cultural beliefs do not influence my decision in taking any vaccine (29-year-old Agnes)*.

*3*.*2*.*2*.*3 Forming judgments on the vaccine*. Participants in the study mentioned what they consider as side effects of the malaria vaccine which they said were experienced by their children. These included the baby crying during the night of the vaccination due to high body temperature but which was seen as normal compared to other previous vaccines received. It was also revealed that these side effects experienced by the babies vaccinated were easily managed by participants.

“*Like the other vaccines we have been taking*, *the malaria vaccine does not have any severe adverse effects*. *I am happy the vaccine will protect my child from malaria*. *After the injection*, *the only thing I noticed was that the child’s body was hot but it went away after giving the child paracetamol syrup” (35-year-old Lariba)*.

Not all participants were of the view that the malaria vaccine protects children fully. Some were of the view that despite the vaccination against malaria, the children are still vulnerable and additional measures are needed to protect them.

“*Yes*, *my child still had malaria after taking the vaccine and so they can still get it*. *The health workers told us this and asked that we should still sleep under mosquito nets and clean our environment*. *But for me what I noticed is that when my child had malaria it was not severe” (22-year-old Jane)*

“*It is possible that vaccinated children can still get malaria if their mothers do not use mosquito nets*, *sprays*, *and coils” (26-year-old Lamisi)*

“*It is possible for vaccinated children to still get malaria if they are exposed to mosquitoes*. *So*, *it is advisable to continue using mosquito repellant*, *nets*, *and sprays to adequately protect the child” (35-year-old Alberta)*.

*3*.*2*.*2*.*4 Benefits of vaccinating a child*. Participants spoke about the benefits of vaccinating their children against malaria. These benefits could be financial in terms of not wasting money to buy drugs or wasting working hours in visiting the hospital to seek medical care.

“*When my child is sick and you go on admission*, *you buy drugs*, *you take care of other things so when they vaccinate and all that stops*, *it will increase the money available that would have been used on drugs and hospital admission*. *Those monies will be used to do something that will make the family better” (34-year-old Anita)*.

“*Now I don’t attend hospital often and the little money I have is not wasted on buying drugs and paying hospital bills*. *These days the health insurance is not even working and if not for the vaccine and my child were to be falling sick like the other ones*, *I don’t know what I will have done” (22-year-old Jane)*

“*Vaccinating my child saves me time and money*. *I concentrated at my workplace and I was not running around as before to get treatment for my sick child” (38-year-old Asana)*.

#### 3.2.3 Reaction to the malaria vaccine

The issue of acceptance of the malaria vaccine was explored and this generated the theme “Reaction to vaccine”. Data from the study indicate that participants were particularly motivated to vaccinate their children seeing the positive effects they believe the vaccine had on their children’s health. Two sub-themes “Motivation to vaccinate child” and “Concerns about vaccine administration” emerged from this theme.

*3*.*2*.*3*.*1 Motivation to vaccinate a child*. Participants recounted several factors that served as a motivation to vaccinate their children, ranging from inspiration from healthcare workers and belief that the vaccine might be responsible for the healthy state of their children. This was evident in their quotes when participants said;

*I will forever vaccinate my child against malaria because when my child was vaccinated, no severe effects were experienced. She looks fine and healthy perhaps because of the vaccine. So, I will always vaccinate my child against malaria (29-year-old Agnes)*.

*We were informed at the health center before allowing our children to be vaccinated and the nurse’s presentation on the vaccine shows that it was safe. My friend who is a nurse also vaccinated her child (27-year-old Jemimah)*.

*3*.*2*.*3*.*2 Concerns about vaccine administration*. In as much as participants had positive things to say concerning the malaria vaccine, they presented some concerns which could have a significant influence on decision-making to vaccinate their children and inspire others to do the same. Some participants were of the view that the mode of administration could affect the vaccine’s efficacy, and the side effects experienced by different children could affect recommending the vaccine to other caregivers.

*Maybe if the vaccination is not done properly or if the vaccines are not effective enough*, *they might still get malaria after vaccination*. *So*, *the nurses should take their time to administer the vaccination safely and properly’ (35-year-old Lariba*).

*Well, it will be difficult for me to encourage other mothers to vaccinate their children*

*because the adverse effects might be severe in some children and I will not like to see*

*any child to be a victim (26-year-old Lamisi*).

## 4. Discussion

This study had the focus of exploring the perceptions and acceptance of the malaria vaccine by caregivers. The findings indicate that the caregivers received information and gained knowledge on malaria and the malaria vaccine for children under-five years mainly through the nurses at the vaccination centers. In other studies [21, 25], awareness on malaria is high among caregivers, similar to findings of the current study but the main source of information on the malaria vaccine was the media [21]. The differences in these findings is because participants in the current study are caregivers with children in a malaria vaccine pilot programme compared to previous studies [21, 25] where administration of the vaccines had not started.

Participants had positive perceptions of the vaccine, which was influenced by their experience with the malaria vaccine program. Their children benefiting from the malaria vaccine programme coupled with the education from the healthworkers, afforded them a first-hand experience with the vaccine to enable them gain insight and be able to form judgment on its benefits, safety and possible side effects among others. Findings from a review of studies by Onyekachi, et al. [25] shows similar positive perceptions on malaria vaccine by participants in the various studies they reviewed. However, children of participants from these previous studies [25] were not taking the vaccines and their perceptions were based on the perceived severity of the disease and its devastating effects and the perception that the vaccine will prevent malaria. This supports the assertion that caregivers or mothers are likely to make decisions regarding vaccination based on perceived risks of contracting the disease, and the perceived effectiveness of the vaccine [26]. Living in a malaria-endemic area like the study area, coupled with education from health workers on the disease, the caregivers were familiar with the severity of the disease. As reported by the participants, the cost associated with their children having malaria goes beyond the suffering of the child. The financial burdens, loss of productive work hours, and the psychological stress of taking care of a sick child affect their peace of mind. Some previous studies show that the cost associated with the treatment of malaria and lost labor in sub-Saharan Africa, is estimated at 12 billion dollars (USD) every year [27–29].

Caregivers narration of their reaction to the vaccination of the children, demonstrated their acceptance of the malaria vaccine though some raised concerns about the vaccine. Participants’ motivation for the vaccination was the perceived advantages over the disadvantages after they weighed several factors in addition to the education they received from the nurses. This finding is consistent with studies conducted by Ojakaa et al. [30] where caregivers decided to vaccinate their children after identifying the perceived long-term advantages of the malaria vaccine. The influence of the education provided by the healthworkers on the positive decisions made by the participants in accepting the vaccines is noteworthy. This contributes to bring to light the importance of public health programs in raising community awareness as reiterated by other studies [30–32]. Several years before the introduction of the vaccine, Ghana was one of the countries in which the Program for Appropriate Technology in Health (PATH) undertook projects to assess community perceptions of vaccines and malaria [33]. The findings supported the development of communication plans and the necessary materials for informed decision-making in vaccine acceptance. Some of these actions could have influenced the vaccine acceptance in communities in which they are being implemented and this needs to be sustained.

Although the participants embraced the benefits of the vaccines, some were not comfortable in recommending this to other caregivers due to possible side effects of the vaccine which they feared some children may experience and their credibility could be at stake. Fears related to vaccine safety, lack of information, ignorance and general knowledge even among healthcare workers have been identified as barriers to accepting vaccines [25, 34, 35]. The concerns expressed by some participants in the current study about the vaccine were based on the side effects experienced by some of the children. This fear of the unknown is a significant factor that requires consideration and the necessary actions to be taken by stakeholders to mitigate it.

### 4.1 Strengths and limitations

The findings of the study are limited to the views from only caregivers whose children were taking the malaria vaccine. Nonetheless it provides valuable information and affords the Ministry of Health and stakeholders especially in malaria endemic areas, an opportunity to plan and address concerns prior to the country-wide roll-out of the programme. It will help in planning strategies to improve caregivers’ perceptions and acceptance of the malaria vaccine.

## 5. Conclusion

Caregivers’ awareness of the devastating effects of malaria in children under-five years, their perceptions, acceptance, and confidence in proven interventions are important in effectively rolling out such interventions. Most of the participants had good knowledge of the malaria vaccine and their experience contributed to their positive perceptions about the vaccine. Fewer hospital admissions, more time to engage in income-generating activities, saving money, and having peace of mind are notable benefits derived from taking a malaria vaccine and education provided by healthworkers contributed to the acceptance of the vaccine However, there are hesitations in recommending the intervention to other caregivers due to fear of the unknown concerning the vaccine’s side effects. To mitigate this, health workers, especially nurses working at the Child Welfare clinics should intensify education on malaria and the malaria vaccine, as well as other public health interventions like the use of insecticide-treated nets, diagnosis and seeking early treatment. This will help build public confidence and facilitate the uptake of the vaccines as countries prepare to roll-out full implementation of the programme.
